# Targeting elimination of cervical cancer by 2030: a baseline assessment in six African countries—part II

**DOI:** 10.3332/ecancer.2022.1454

**Published:** 2022-10-07

**Authors:** Daniela Cristina Stefan, Jean-Marie Dangou, Prebo Barango, Issimouha Dille Mahamadou, Sharon Kapambwe

**Affiliations:** 1 Institute of Global Health Equity Research Rwanda, University of Global Health Equity, 0000 Kigali, Rwanda; 2 World Health Organization Regional Office for Africa, P.O. Box: 06, Brazzaville, Republic of Congo

**Keywords:** cancer control, WHO targets 2030, Africa, health personnel, training, pathologists

## Abstract

**Background:**

Africa is home to many countries with the highest incidence of cervical cancer in the world. It is encouraging to see that the efforts to prevent and manage this disease are intensifying across the continent. The World Health Organization (WHO) has, in the last years, led a world-wide initiative to eliminate cervical cancer as a public health problem, starting by setting clear targets for 2030.

**Aim:**

To assist those African countries with the largest burden of cervical cancer, to reach the set targets, as a first step, WHO initiated a baseline capacity assessment in African high burden countries. We present and discuss the results thereof in this paper.

**Method:**

The countries selected for the baseline evaluation were Eswatini, Guinea, Malawi, Rwanda, Uganda and Zambia. The data were collected by a mailed questionnaire in English, with 129 questions, most of them with preset answer options. It was answered by national coordinators of non-communicable diseases, cancer control or reproductive health programmes in the ministries of health and by the WHO country representatives. Based on the answers, suggestions were made towards optimising the efforts for cancer control.

**Results:**

Except for Eswatini and Guinea, vaccination against the human papilloma virus (HPV) has reached between 74% and 98% of girls in the age bracket. The main method for cervical screening is still inspection with acid acetic. HPV testing, recommended by WHO, is being introduced slowly. The numbers of women screened are not yet nearing the required levels. Between 30% and 70% of the cervical cancers diagnosed are being treated with palliative intent. A deficit of personnel was reported for all professions involved in cervical cancer care.

**Conclusions:**

Guinea will need assistance to implement HPV immunisations and expand screening. In all six countries surveyed, steps should be taken to introduce or scale up the more precise HPV screening instead of acid acetic inspection of the cervix, to replace the current cryoablation of the preinvasive lesions of the cervix with thermal ablation and to increase the capacity for screening. Solutions need to be found for covering the dearth in gynaecological oncologists and radiotherapy installations and personnel.

## Introduction

Cancer of the cervix was the world’s fourth most frequent cause of death from malignant disease in women in 2020 [1]. What distinguishes it from many other cancers is that most cases are due to a chronic infection with human papilloma virus (HPV), and as such it is almost completely preventable by vaccination of girls [2], combined with screening women at regular intervals to detect and treat it in the pre-invasive stage. Sub-Saharan Africa counted 19 out of the 20 countries in the world with the highest burden of cervical cancer, in 2018 [3].

This is an evaluation of the readiness of six African countries to implement progressive changes leading to the elimination of the cancer of the uterine cervix as a public health problem. As a first step on the path to this global objective, announced in 2018, The World Health Organization (WHO) has set attainable targets for 2030: to vaccinate 90% of girls by 15 years of age against the HPV, to screen 70% of women by 30 years of age and again by 45 years, with a high-performance test, and to treat 90% of pre-invasive cervical cancers, as well as manage 90% of invasive cancers [4].

The African countries selected by WHO for a baseline evaluation, to assist them to develop the required capacity for cervical cancer control: Eswatini, Guinea, Malawi, Rwanda, Uganda and Zambia, constituted a ‘first wave’, with evaluations of other countries to follow. The choice was based on the paucity of actionable information in the available research, which was focusing on isolated aspects of cervical cancer prevention and management. It was decided therefore to obtain a detailed set of data, via the WHO country representatives, on the significant components of the cervical cancer burden, its prevention and management. The findings will be used to devise solutions to assist these countries, as well as other African countries which will be evaluated in the future, to implement and scale up the appropriate solutions on the path to eliminating the cervical cancer as a public health problem.

The data published here contribute to create a clear, comprehensive and true image of the cervical cancer prevention and management situation in these countries. They will be used as baseline to evaluate progress towards the goals set by WHO. They also offer an information platform, on which further research projects may be designed.

## Methodology

The data for this study were collected with a survey by mailed questionnaire in English, with 129 questions, most of them with preset answer options. The questionnaire was answered by national coordinators of non-communicable diseases, cancer control or reproductive health programmes in the ministries of health, as well as by the WHO country representatives. To clarify some answers or obtain information where questions were left unanswered, interviews were held via the Internet with the respondents. Most of the 2020 data on HPV vaccination were extracted from WHO subsequent publications, as they were not available at the time of the survey. The results were grouped in comparative tables for ease of analysis (Tables 1–5).

## Results

### Primary prevention

At the time of data collection, Eswatini and Guinea had not introduced HPV vaccination in their immunisation programmes. Eswatini was not eligible for GAVI support; however, in May this year, the country announced the start of its vaccination programme from September [5, 6]. Guinea has not yet applied [7].

Malawi has introduced HPV vaccination in the national immunisation programme in 2019. It is estimated that 84% coverage of the targeted population was achieved in 2020 [8].

In Rwanda, 98% of the eligible population group was vaccinated during the 2011–2018 period. Presently, the vaccination is administered only to the 12 years age group, with an estimated coverage of 89% [9].

In Uganda, the country-wide roll-out of HPV vaccination started in 2015, with the aim of covering 80% of the target group. The estimated coverage of the targeted population (girls by the age of 15) in 2020 is 74% [10].

Zambia has entered the vaccination in its national immunisation programme in 2019. The estimated coverage with two doses was 70% in 2020 [11].

### Secondary prevention

The prevailing method of screening for cervical cancer is VIA in all six countries, practiced at public health institutions at all levels of care. It is also used in private centres in Malawi and Zambia. Cytology is offered at secondary and tertiary centres in Malawi and Zambia, while in Rwanda and Uganda only at tertiary centres. HPV testing is done in public facilities at all levels in Zambia, and at primary and secondary level in Malawi, if part of research studies. Private units offer HPV testing only in Malawi and Zambia.

The numbers of screening facilities of primary and secondary levels per 100,000 women of 35–49 years of age are varied, from 1.5 in Guinea to 7.6 in Uganda to 13.6 in Rwanda.

Colposcopy, biopsy and histology are offered mainly in tertiary hospitals. At secondary level, colposcopy and biopsy are available at some units in Malawi, Rwanda and Uganda. Some private and NGO units are offering these services in Eswatini, Guinea, Rwanda and Zambia, while for the other countries the information on private facilities is missing.

Cryotherapy is widely used for treating precancerous lesions at all three levels of public facilities, mostly in a screen-and-treat setting. Thermal ablation is used in secondary units in Guinea, Malawi and Zambia, and in all tertiary units except in Eswatini. Tertiary centres also practice Large Loop Excision of the Transformation Zone (LLETZ), cervical conization and hysterectomy. There is little information on the services offered by private centres or by other non-governmental facilities.

### Tertiary prevention

Only Uganda and Zambia offer all therapeutic modalities for invasive cancer. However, while in Zambia around 70% of patients are treated with curative intent, in Uganda the figure is only 30%–40%. As the waiting time from diagnosis to beginning of therapy is just 1 month in Uganda, the difference may be due to a massive late-stage presentation of patients in this country.

It is notable that radical hysterectomy is not offered in Eswatini and Rwanda, indicating the need to train gynaecologists in those countries.

Finally, neither radiotherapy nor brachytherapy is available in Eswatini, Guinea and Malawi. Brachytherapy is also not available in Rwanda.

## Discussion

### Primary prevention

The vaccination of pre-adolescent girls against the HPV at the low price per dose negotiated by Gavi is a highly cost-effective method of prevention of the cervical cancer in the countries surveyed [12, 13]. Recent comparative studies of cohorts of vaccinated and unvaccinated girls have shown up to 87% reduction in the cancer rates in the vaccinated group, compared with the unvaccinated population [2, 14]. The remarkable performance achieved in Rwanda – of immunising 98% of the target population with at least one dose between 2011 and 2018, and of continuing to achieve an estimated 89% coverage in 12 years old girls through 2020 [9] – may constitute an useful case study. Rwanda’s success was based on i) government ownership and support; ii) vaccinating children while at school; iii) broad social mobilisation and iv) adequate strategies for reaching girls not attending school [15].

A potentially highly effective tool might be included in the vaccination programme: mobile phone messaging, driven by the e-health teams at the ministries of health, could be devised within a short time, reach a large audience and contribute to mobilising parents to bring their daughters to be immunised. The messages could offer to the parents, at a minimum, the essential information for understanding the threat of cervical cancer, its origin in the chronic infection with HPV, the benefits of vaccination and information on the age group targeted, as well as on the location and opening hours of the vaccination centres.

Another important success in primary prevention of cervical cancer was registered this year in Eswatini. The country is on the list of those with highest incidence of the disease in the world [3], associated with an estimated total yearly cost of 19 million USD [16]. This year, in September, the planned vaccination of 80,000 girls is due to start.

There is very little available information on the state of prevention of cervical cancer in Guinea. A study of 831 women from Conakry [17], published in 2009 found a 47.9% prevalence of HPV in cervical samples of 752 women without any cervical lesion detected, and of 78.5% in samples from 79 women presenting abnormal cytological findings. In the whole study group, half of women reported having had two or more sexual partners, and about two-thirds indicated that their husbands were polygamous or had extramarital affairs.

The age-standardised incidence rate of cervical cancer in 2021 was estimated to be 50.1 [7]. Guinea does not have a HPV vaccination plan and the cervical cancer screening covers around 10% of women aged 30–49 years [7]. Only about 21% of the population knows about the existence of a vaccine against cervical cancer [18]. The above findings, even if incomplete, recommend Guinea for immediate support towards improving its prevention and management of cervical cancer.

Apart from HPV vaccination, condom usage and circumcision, advised on a populational scale for reducing the spread of HIV, have a complementary benefit of significantly preventing the transmission of HPV [19], and information on this additional effect could be inserted into the educational messages for prevention of HIV-AIDS. There are also other possibilities of exploiting the synergies between cervical cancer prevention and HIV prophylactic or therapeutic activities, both for education and screening.

### Secondary prevention

Our data confirm that the visual inspection of the cervix after application of acetic acid (VIA) is the main method of screening for dysplasia of the cervix in the countries surveyed, even in tertiary level and private care institutions. HPV testing, strongly supported by WHO as being more accurate than VIA, is not yet widespread. In Zambia, HPV screening is offered at all levels of public healthcare as well as in private healthcare. It is estimated that some 10% of screenings will be done by this method in 2023. The cost of screening one person with HPV detection is 23 USD, i.e. ten times higher than the cost of VIA. Zambia’s experience in HPV testing on a larger scale should be studied, for possible solutions applicable in other African countries [20].

WHO has updated the guidelines for screening and treatment of cervical pre-cancer lesions [21] at the end of 2020, devising seven distinct protocols, to be followed depending on equipment and health personnel skills, with a view to replacing VIA as the primary screening method with a large-scale use of HPV molecular testing. HPV tests have a higher detection rate for CIN (cervical intraepithelial neoplasia) 2+ and CIN 3+, while generating less false-negative results than VIA [22] or cytology [23], properties that support its use as a primary screening method. The on-going improvement of HPV testing will make it progressively more suited for single-visit screen-and-treat sessions.

The planning of infrastructure, material resources, human resources and budget necessary to achieve the screening proposed by WHO needs to consider the – often large – size of the population targeted. To exemplify the magnitude of the effort waiting ahead, we may consider the case of Uganda. By using the population pyramid of Uganda [24] for instance, screening 70% of those women who would have been in their 34th and 44th year of age in 2021, means that around 340,000 women would have had to be screened in 1 year. Considering the continuing growth of the country’s population, even more women per year will need to be screened in the future.

A recent study of HPV prevalence in rural Uganda [25], using an assay for mRNA, found that 21% (95% CI = 19%–23%) of the 1,892 women tested were carrying high-risk HPV; however, the study found HPV-16 and HPV 18/45, alone or in combination, only in 4.6% of the cases. By assuming that the same prevalence of high-risk HPV exists in the entire population, it results that, after having tested for HPV the whole age group mentioned above, around 70,000 triaging VIA would have been performed in 2021, if the assay mentioned were used, or 17,000 VIA if using only an assay for HPV-16 and -18. The present capacity for screening should be substantially augmented to accommodate such demand. Uganda does not have yet a national screening programme. In the last 5 years, only 8% of the female population from 30–49 years of age were screened, and only 10% of that targeted group were ever screened [10].

The planning should also consider the investigations and treatment procedures, like cryo- or thermal ablations, LLETZ and cervical conization histopathology specimens generated by the screening, and the management of the additional invasive cancers discovered. For realistic planning, estimating the prevalence of cervical infection with HPV and its main oncogenic variants, by testing a representative sample of the population targeted, should be done in the countries surveyed, as soon as possible.

An important component of the migration to a large-scale HPV screening would be point-of-care testing, rather than processing the samples in centralised laboratories. As the technology of testing evolves, it may become possible to screen and treat during a single visit.

This survey indicated that cryotherapy is still the most widely used method of cervical dysplasia ablation, at primary and secondary levels of healthcare, in the countries surveyed. The notorious inconvenient of this method is that it is plagued by insufficient stocks of freezing agent. WHO encouraged replacing it by thermal ablation powered by electricity, either from a desktop source or from rechargeable batteries [26]. LLETZ training should be expanded where possible, as it offers the possibility to treat those lesions that are more difficult to manage by other means, and more importantly, it provides sufficient tissue to enable diagnosing early invasive forms of the disease.

### Tertiary prevention

Our data (Table 4) shows that the whole therapeutic arsenal for invasive cervical cancer is available only in Uganda and Zambia. Radical hysterectomies, the treatment of choice for early stages of the disease, are not being performed in Eswatini and Rwanda. This finding draws attention to the general deficit of gynaecological oncologists on the continent, and particularly in Sub-Saharan Africa, where only seven countries offer postgraduate programmes of training in this sub-speciality [27]. This happens while the need for radical hysterectomies is forecasted to increase, because of screening increasingly more women. Some of those will present with early invasive cervical cancers, highly curable by surgery [28].

In three of the countries surveyed, no radiotherapy is available. Rwanda has linear accelerators but no brachytherapy. Some of the patients from countries where no irradiation treatment is possible, are referred to facilities beyond borders, which incurs additional expenses for travel and subsistence, above the cost of treatment. Presumably most of the patients meant only for palliative treatment do not receive any irradiation under the present circumstances. This group constitutes more than half of the newly diagnosed cases, in some of the countries studied.

The solution requires the involvement of governments, in partnership with financial institutions, donors and the African Commission on Nuclear Energy, in drawing and finalising projects for new radiotherapy facilities and personnel recruitment and training. The International Agency for Atomic Energy has established that one linear accelerator can treat around 450 cases of cancer annually, meaning that calculating with an average cancer incidence and distribution of various cancers, five facilities would be necessary for 1 million of inhabitants. According to these norms, Rwanda would need 52 radiation therapy units, Uganda 228 and Zambia 89. Such coverage has not yet been attained on the African continent, but the figures are supported by thorough studies, and constitute a clear target for planning, over a longer perspective [29].

This study also shows discordant numbers of cases diagnosed versus cases treated versus distribution of cases per stages. These reflect a problem with cancer registration and extraction of data. It may be necessary to review the nature of captured data on cervical cancer, to ensure that they provide adequate information for planning, and digitise the recording and storing of the respective information.

A few data on palliative management have been collected. The relatively small numbers of palliation specialists reported are contrasting with the significant percentages of cases managed with palliative intent. Out of the six countries studied, only Uganda reported that specialised accredited training in palliation is available. However, the existence of primary healthcare personnel trained in morphine administration is documented only in Eswatini; no data were received from the other countries, although the literature mentions the existence of home palliative care services [30].

The consumption of morphine (equivalent) is reported in milligrams per capita, for ease of comparison. A large discrepancy exists between the figures reported and published data from high-income countries: in the countries surveyed, the morphine use varies from less than 1 mg per capita to 3 mg; the literature shows consumptions of 241 mg per capita in UK, 213 mg in France and 204 in Italy [31].

### What is new in this study?

This report contains information provided by the national coordinators of various governmental institutions and programmes whose purpose is to control the cancer of the cervix, in the six countries surveyed. Together with its complementary report [32], it depicts a comprehensive and accurate image of the actual state and future needs of the health systems, health facilities and health personnel providing prevention and treatment, at the time of the survey. To the authors’ knowledge, data of similar quality and depth, coming from Africa, are rarely published.

Our data highlight truthfully the progress of HPV immunisation in the countries studied, the challenging dimensions of the cervical cancer screening lying ahead and the need to urgently train or acquire more pathologists, surgical and radiation oncologists and to build more radiotherapy facilities. They clearly indicate that strong leadership, international cooperation and innovative solutions are needed for protecting African women and their families from this disease, which is at present highly preventable and curable.

### What are the shortcomings of this study?

We could only formulate a few reasonable and realistic suggestions towards addressing the problems we identified. We are highly aware that only someone that is directly and permanently leading health care activities at various levels would be able to find adequate and effective answers. However, our synthesis may help to formulate strategies and to better prepare for the challenges ahead, going towards the elimination of cervical cancer as a public health problem. Of particular interest in this respect is the experience of Zambia, where HPV testing is being offered at all public facilities. A future analysis of that activity might extract useful lessons for the introduction or scaling up of HPV screening in other African countries. Unfortunately, we did not obtain data on the number of facilities already offering HPV screening in the countries surveyed.

Another shortcoming of the analysis of secondary prevention activities was that the actual coverage of the target population through opportunistic screening is not known; the figure is necessary for estimating the necessary escalation of the service to reach the 2030 target. It remains to be determined or estimated by the health authorities in each country.

## Conclusions

Four of the six countries surveyed have active vaccination programmes. Guinea should be supported towards starting her own vaccination programmes.

The migration from the almost ubiquitous VIA primary screening method to HPV testing will happen gradually. The main advantage of a negative HPV test is that one can be highly confident that the patient can be recalled according to the appropriate testing schedule without risk. The main disadvantage right now is that if a treatment of dysplasia is needed, the patient must travel for a second visit. However, techniques of self-sampling may obviate the need for many visits at the health centres.

Scaling up the screening and its associated medical interventions will require many more testing points and personnel but will be rewarded by a reduction in the costs of cervical cancer care and substantially improved survival rates. To improve the numbers of patients treated with curative intent, it is the responsibility of governments to plan and budget for the training of the necessary specialists in gynaecologic oncology and for establishing radiotherapy facilities.

However, a large proportion of the diagnosed cases is managed at present with palliative intent, due to late presentation, but also because of limited capacity to perform radical hysterectomies or radiotherapy, in those cases where they would be indicated. The patients in palliative care receive limited pain therapy, judging from the consumption of morphine equivalent per capita. We should not neglect the suffering of the many patients who presently are beyond cure, while working to improve our curative capabilities.

To monitor the progress towards the 2030 targets set by WHO, the cancer registries should be improved, both with regard to data collection and ease of data retrieval and analysis.

## Funding

None.

## Conflicts of interest

None declared.

## Figures and Tables

**Table 1.** Number of cervical cancer screening facilities and screening method employed.

Country			Eswatini^a^	Guinea^a^	Malawi	Rwanda	Uganda	Zambia
Public facilities	Primary	Number	125		218	61	159	34
		Cytology			-	-	-	Yes
		VIA^b^			Yes	Yes	Yes	Yes
		VILI^c^			-	-	-	-
		HPV			-	Yes	-	Yes
	Secondary	Number	7	13	28	28	-	106
		Cytology			Yes	Yes	-	Yes
		VIA			Yes	Yes	Yes	Yes
		VILI			Yes	Yes	-	-
		HPV		0	-	-	-	Yes
	Tertiary	Number	5	2	4	5	15	5
		Cytology			Yes	Yes	Yes	Yes
		VIA			Yes	Yes	Yes	Yes
		VILI			Yes	-	-	-
		HPV		0	-	-	-	Yes
Private		Number	8	2	N/A	4	-	5
		Cytology			Yes	Yes	Yes	Yes
		VIA			Yes	-	-	Yes
		VILI			Yes	-	-	-
		HPV			Yes	-	-	Yes

a Data are missing for these countries

b VIA, Visual inspection after application of acetic acid

c VILI, Visual inspection after application of Lugol iodine

N/A, None

**Table 2.** Number of facilities providing diagnostic of cancerous lesions.

Level of care		Service	Eswatini	Guinea	Malawi	Rwanda		Uganda	Zambia
Public	Primary	Colposcopy	0	0	0	0	0		0
		Biopsy	0	0	0	0	0		0
		Histology	0	0	0	0	0		0
	Secondary	Colposcopy	0	0	Yes^a^	14	14		0
		Biopsy	0	0	Yes^a^	7	14		41
		Histology	0	0	0	0	0		2
	Tertiary	Colposcopy	1	2	4	4	3		1
		Biopsy	1	4	4	5	3		4
		Histology	1	2	3	5	1		3
Private		Colposcopy	Yes^a^	1	N/A	2	N/A		1
		Biopsy	Yes^a^	1	-	4	-		14
		Histology	Yes^a^	0	-	1	-		5
Other		Colposcopy	0	1	N/A	N/A	N/A		-
		Biopsy	0	1	-	-	-		1
		Histology	0	0	-	-	-		-

a Number not available

**Table 3.** Facilities providing treatment of precancerous lesions.

Level of care		Service	Eswatini	Guinea	Malawi	Rwanda		Uganda	Zambia
Public	Primar	No facilities	137	0	104	61	7		30
		Cryotherapy	Yes	-	Yes	Yes	Yes		Yes
		Thermal ablation	-	-	-	-	-		-
		LLETZ^a^	-	-	-	-	-		-
		Conization	-	-	-	-	-		-
		Hysterectomy	-	-	-	--	-		-
	Secondary	No. facilities	7	13	28	4	13		116
		Cryotherapy	Yes	Yes	Yes	Yes	Yes		Yes
		Thermal ablation	-	Yes	Yes	-	-		Yes
		LLETZ	-	Yes	-	-	-		-
		Conization	-	Yes	-	-	-		-
		Hysterectomy	-	No	-	-	-		-
	Tertiary	No. facilities	5	3	4	4	3		5
		Cryotherapy	Yes	Yes	Yes	Yes	Yes		Yes
		Thermal ablation	No	Yes	Yes	Yes	Yes		Yes
		LLETZ	Yes	Yes	Yes	Yes	Yes		Yes
		Conization	Yes	Yes	Yes	Yes	Yes		Yes
		Hysterectomy	Yes	Yes	Yes	Yes	Yes		Yes
Private		No. facilities	8	2	N/A	0	N/A		N/A
		Cryotherapy	Yes	Yes	-	-	-		-
		Thermal ablation	N/A	Yes	-	-	-		-
		LLETZ	N/A	Yes	-	-	-		-
		Conization	N/A	Yes	-	-	-		-
		Hysterectomy	N/A	Yes	-	-	-		-
Other		No. facilities	3	N/A	N/A	0	N/A		1
		Cryotherapy	Yes	-	-	-	-		N/A
		Thermal ablation	N/A	-	-	-	-		N/A
		LLETZ	N/A	-	-	-	-		N/A
		Conization	N/A	-	-	-	-		N/A
		Hysterectomy	N/A	-	-	-	-		N/A

a Large loop excision of the transformation zone

**Table 4.** General information related to invasive cervical cancer treatment.

Country		Eswatini	Guinea	Malawi	Rwanda	Uganda	Zambia
Services offered	Hysterectomy	Yes	Yes	Yes	Yes	Yes	Yes
	Radical hysterectomy	No	Yes	Yes	No	Yes	Yes
	Chemotherapy	Yes	Yes	Yes	Yes	Yes	Yes
	Radiotherapy	No	No	No	Yes	Yes	Yes
	Brachytherapy	No	No	No	No	Yes	Yes
	Other/specify	No	No	No	No	No	N/A
At what level	Primary	-	-	-	-	-	-
	Secondary	-	-	-	-	-	Yes
	Tertiary	Yes	Yes	Yes	Yes	Yes	Yes
	National referral hospital	Yes	Yes	Yes	Yes	Yes	Yes
Nr. of cancer centres/hospitals		1	5	2	2	2	2
Time from diagnostic to treatment (months)		6	5–6 weeks	3	1	1	3
Nr. of diagnosed with cervical cancer/year (FIGO staging)	I	6	76	N/A	16	1,203	99
	IIA	58	267	N/A	12	1,456	140
	IIB			N/A	44	1,867	
	III	N/A	268	N/A	109	1,645	207
	IV	20	154	N/A	30	1,062	406
All stages		480^a^	765	4,163	351^a^	6,413^a^	1,800^a^
Nr. patients receiving treatment per year	Curative intent	80^b^	140^b^	N/A	321	30%–40%	70%
	Palliative	N/A	420^g^	N/A	30	60%–70%	30%

a Stages not recorded for all patients

b A number of patients were treated elsewhere

FIGO: International Federation of Gynecology and Obstetrics

**Table 5.** Data on palliative management of cervical cancers.

	Eswatini	Guinea	Malawi	Rwanda	Uganda	Zambia
Standardised referral	No data	No data	Yes	Yes	Yes	No
Palliation specialists	1	None	No data	5	No data	3
Managed with palliative intent	No data	75%	No data	10%	Over 50%	30%
Morphine^a^	2 mg	0	3 mg	<1 mg	<1 mg	<1 mg

a Oral morphine equivalence per capita, 2017 [6–11]
